# Ciliary Membrane Lipid Homeostasis in Health and Disease

**DOI:** 10.1002/advs.77971

**Published:** 2026-09-28

**Authors:** Zhenzhou Huang, Yuan‐yuan Liu, Hanqi Sun, Peiwei Liu

**Affiliations:** ^1^ College of Life Sciences Shandong Normal University Jinan China

**Keywords:** BBS, cholesterol, ciliary lipid, ciliary membrane, ciliopathy, IFT

## Abstract

The ciliary membrane is an active, highly specialized lipid landscape that dictates ciliary identity and function. Distinct from the surrounding plasma membrane, it exhibits a tailored lipid architecture—enriched in specific phosphoinositides, cholesterol, and sphingolipids—that shapes membrane curvature and establishes spatial platforms for signaling cascades. Emerging evidence indicates that disrupting the lipid microenvironment gives rise to a distinct spectrum of ciliopathies, driven by mislocalized signaling receptors, impaired intraflagellar transport (IFT), and disintegrated inner structures. This review bridges biophysical membrane dynamics with genetic pathology, positioning ciliary lipids not merely as structural components, but as active orchestrators of ciliary gatekeeping, cilia motility, and cilia signal transduction. By remapping the molecular mechanics of lipid dysregulation in diseases such as Bardet‐Biedl syndromes, we highlight how targeting the ciliary lipidome opens new therapeutic avenues for human ciliopathies.

## Graphical abstract

This review systematically delineates the distinct lipid landscapes of ciliary membranes, including the spatial organization of phosphoinositides, cholesterol, and sphingolipids. It elucidates how these lipids orchestrate ciliogenesis, signal transduction, and membrane dynamics in cilia beating, and highlights how their dysregulation drives ciliopathies such as Bardet‐Biedl and Joubert syndromes. Discussing emerging lipid‐targeted therapies, we position ciliary lipids as central mediators of disease pathogenesis and promising drug targets.

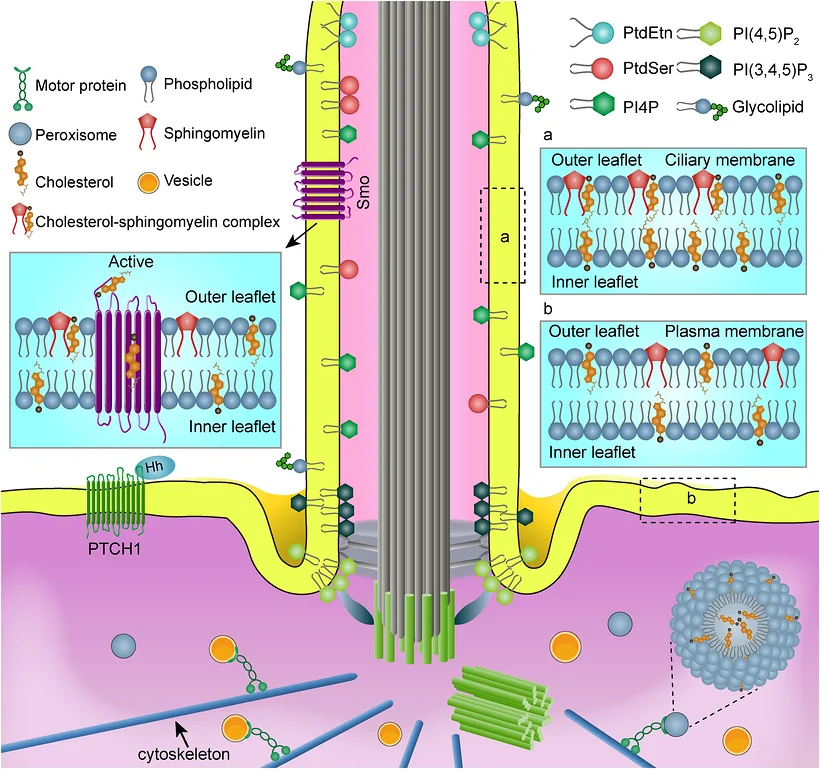

**Abbreviations**
7β,27‐DHC: 7β,27‐dihydroxycholesterol
BBS: Bardet‐Biedl syndrome
GlcCer: glucosylceramide
IFT: intraflagellar transport
LacCer: lactosylceramide
PA: palmitic acid
PI(3,4,5)P_3_
: phosphatidylinositol 3,4,5‐trisphosphate
PI(4,5)P_2_
: phosphatidylinositol 4,5‐bisphosphate
PI4P: phosphatidylinositol 4‑phosphate
POA: palmitoleic acid
PtdCho: phosphatidylcholine
PtdEtn: phosphatidylethanolamine
PtdIns: phosphatidylinositol
PtdSer: phosphatidylserine

## 1 Introduction

Cilia, also known as flagella, are highly conserved hair‐like organelles on the surface of eukaryotic cells. Cilia are enclosed by a lipid bilayer membrane that is continuous with the plasma membrane, while the ciliary lumen is continuous with the cytoplasm. The internal axoneme is composed of microtubules and associated proteins [1]. The anchoring end of the axoneme, which is located in the cytoplasm, is termed the basal body and is typically derived from the mother centriole. The transition zone (TZ), a highly ordered structure positioned above the basal body together with its associated structure, forms the ciliary gate and acts as a selective molecular sieve that strictly controls the passage of substances into and out of the cilium [2, 3]. Despite decades of research, most studies have focused on the protein components of cilia. By comparison, the ciliary membrane and its unique lipid composition have long been underappreciated.

The ciliary membrane, which remains largely underinvestigated, plays an indispensable role in the normal physiological functions of cilia. Its lipid composition exhibits distinct features that accommodate ciliary structure and function, distinguishing it from the plasma membrane. Major components include phospholipids such as phosphatidylcholine (PtdCho), phosphatidylethanolamine (PtdEtn), phosphatidylserine (PtdSer), phosphatidylinositol (PtdIns), cholesterol, and sphingolipids (sphingomyelin, glycosphingolipids, ceramides) (Figures 1 and 2) [4, 5]. In mammals, the lipid composition of primary cilia (from Madin‐Darby canine kidney (MDCK) cells) and motile cilia (from *porcine* olfactory epithelium) has been analyzed by mass spectrometry following cilia isolation [6, 7]. The sterol profile of primary cilia shows enrichment of cholesterol and desmosterol [6], whereas motile cilia lack cardiolipin, exhibit reduced PtdEtn, and are enriched in sulfoglycosphingolipids [7]. This functional distinction likely reflects adaptive specialization: primary cilia enrich sterols to bolster membrane microdomains for signal transduction, whereas motile cilia modulate phospholipid and sphingolipid composition and distribution to optimize beating dynamics and membrane resilience. However, a direct lipid comparison between the two cilia types is still lacking, and existing studies have mostly focused on specific classes, such as sterols or phospholipids, without establishing a comprehensive lipidomic map of mammalian ciliary membranes or exploring the functions of lipids. Because intact cilia can be isolated and purified from unicellular organisms, their lipid composition has been further investigated and shows diversity across species. In *Trypanosoma brucei*, the ciliary membrane contains higher levels of PtdEtn, PtdSer, ceramides, and sphingomyelin than the whole cell, whereas PtdCho and PtdIns are severely deficient [8]. *Paramecium* cilia exhibit elevated phospholipids, sphingolipids, and polyunsaturated fatty acids [4]. Studies on *Chlamydomonas reinhardtii* cilia showed that the ciliary membrane is enriched in phytoceramide, ergosterol, and PtdEtn, with a high content of saturated short‐chain fatty acids that contributes to the maintenance of ordered lipid microdomains, such as the ceramide‐enriched compartment at the ciliary base [9, 10]. These differences highlight the adaptive characteristics of ciliary membranes in unicellular organisms, for example, lipids for photophysical process, such as phytoceramide [9, 11], may tend to accumulate in *Chlamydomonas* cilia.

**FIGURE 1:**
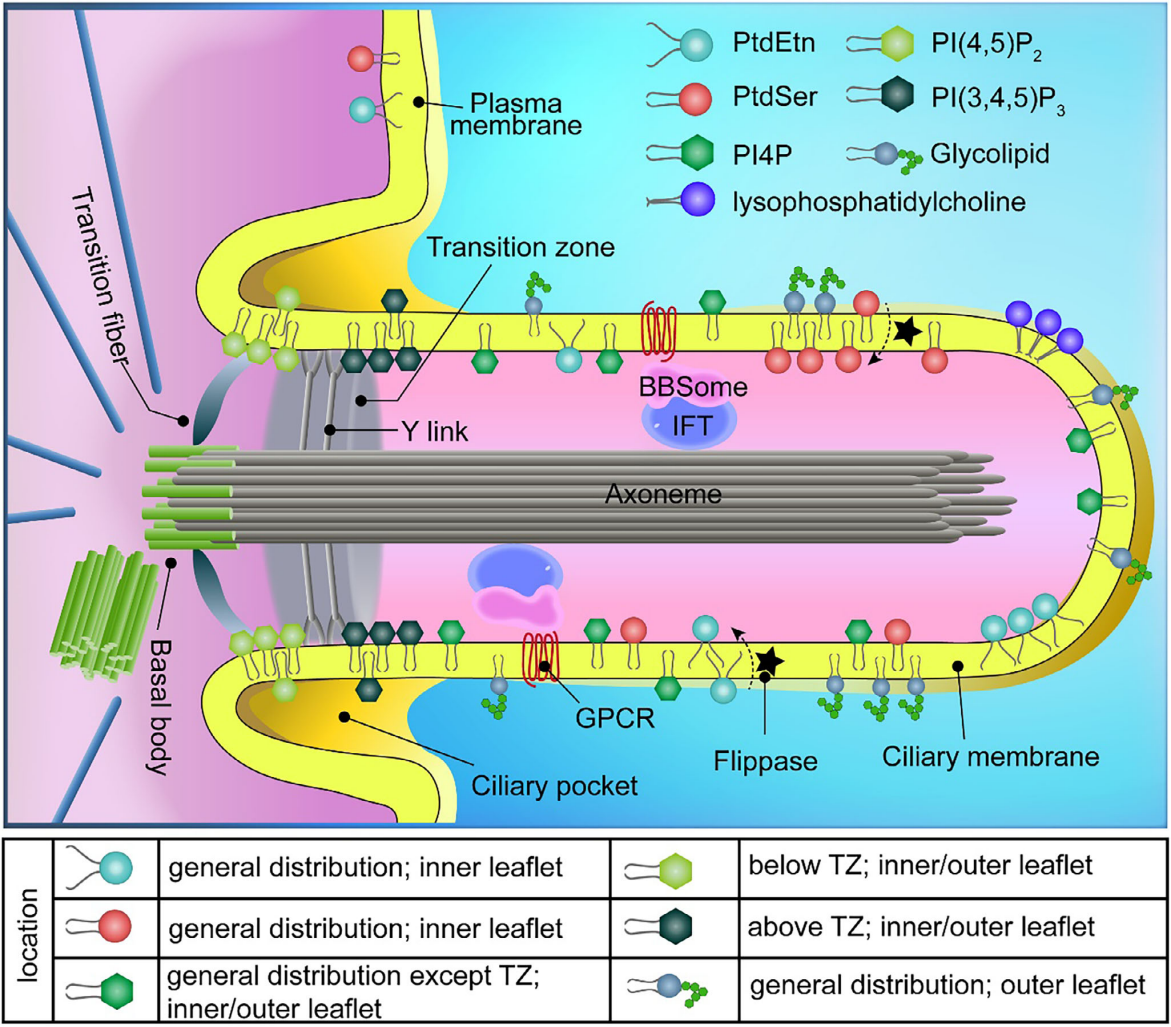
Schematic representation of cilium structure and ciliary lipid distribution. Schematic overview of the major structural components of the cilium, including the basal body, TZ, axoneme, and ciliary membrane, together with the distribution of major lipid classes. The TZ is positioned above the basal body and functions as a selective ciliary gate. Y‐shaped linker structures within the TZ connect the axoneme to the ciliary membrane. The ciliary membrane has a lipid composition distinct from that of the surrounding plasma membrane. PtdEtn and PtdSer are predominantly localized to the inner leaflet of the ciliary membrane, where their asymmetric distribution is maintained, in part, by flippases in certain organisms like *Chlamydomonas*. Lysophosphatidylcholine is present mainly at the outer leaflet to assist in maintaining the curvature of the tip membrane. PtdIns and its phosphorylated derivatives exhibit specific distributions within the cilium, with PI(4,5)P_2_ and PI(3,4,5)P_3_ primarily associated with the TZ and PI4P enriched in regions distal to the TZ. Glycolipids are predominantly localized to the outer leaflet, with their carbohydrate moieties exposed to the extracellular environment and contributing to membrane organization and cell‐surface recognition. The distribution of the major phospholipid and glycolipid classes is summarized in the accompanying table. In the table, the term before the semicolon indicates the ciliary subregion, whereas the term after the semicolon indicates localization to the inner or outer leaflets of the ciliary membrane.

**FIGURE 2:**
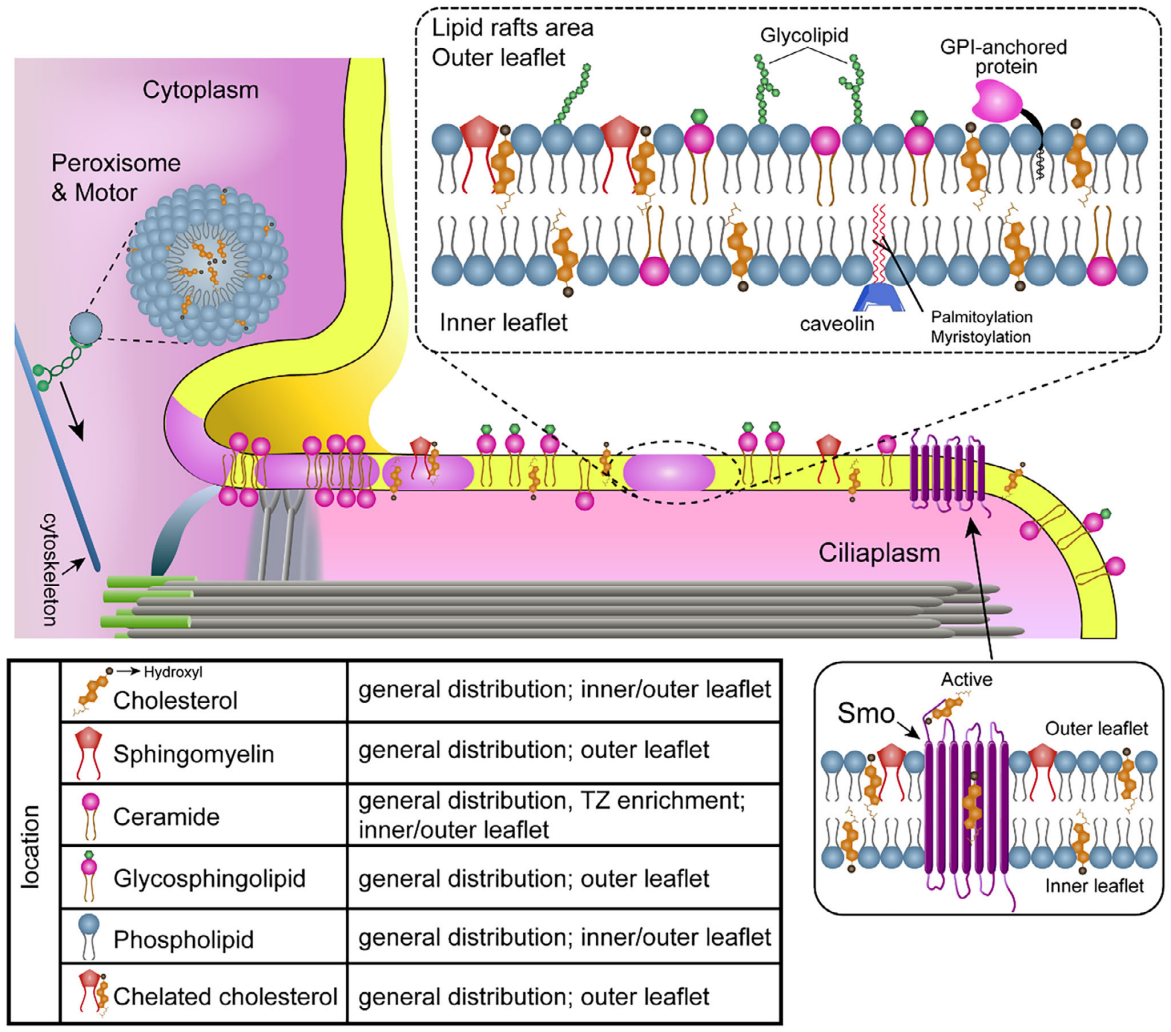
Distribution of cholesterol/sphingolipids and microdomains (lipid rafts) in the ciliary membrane. Schematic representation of the distribution of cholesterol and major sphingolipid classes, including sphingomyelin, glycosphingolipids, and ceramides in the ciliary membrane. Cholesterol is an amphipathic sterol containing a hydrophobic steroid ring and a hydrophilic hydroxyl group. Cholesterol participates in cilia‐based signaling pathways, including Hh signaling. Additionally, cholesterol can be chelated by sphingomyelin. Sphingomyelin is predominantly localized to the outer leaflet of the ciliary membrane and can associate with cholesterol to form sphingomyelin‐cholesterol‐rich membrane domains, which are also known as lipid rafts. Glycosphingolipids, consisting of a ceramide backbone linked to carbohydrate chains, are predominantly localized to the outer leaflet. Ceramides are distributed throughout the ciliary membrane and are especially enriched in the TZ, with localization in both membrane leaflets. The dashed‐line box illustrates the organization of lipid rafts within the ciliary membrane. These cholesterol/sphingolipid‐enriched domains are particularly prominent at the ciliary base and TZ. Lipid rafts accumulate GPI‐anchored proteins and proteins modified by lipidation, including palmitoylation and myristoylation, thereby providing membrane platforms for the organization and regulation of signaling proteins. In the table, the localization of the major sphingolipid classes is summarized. The term before the semicolon indicates the ciliary subregion, whereas the term after the semicolon indicates localization in the inner or outer leaflets of the ciliary membrane.

In this review, we summarized the specific distribution of lipids in the ciliary membrane, the roles of lipids in cilia assembly and disassembly, cilia motility, as well as cilia signaling pathways, and sought to provide new insights into the abnormal lipid functions in diseases.

## 2 Cilia Membrane Lipids

### 2.1 Fatty Acid Metabolism and Ciliogenesis

Abnormal fatty acid metabolism alters fatty acid availability, leading to the mislocalization of ciliary proteins or lipids, which affects ciliogenesis or cilia functions. A high‐fat diet upregulates stearoyl‐CoA desaturase 1 (SCD1), which converts palmitic acid (PA) to palmitoleic acid (POA), thereby depleting cytoplasmic PA, reducing the palmitoylation of ADP‐ribosylation factor‐like protein 13B (ARL13B), Ras‐related protein Rab‐8A (RAB8A), and centrosomal protein 131 (CEP131), compromising their stability and localization, and ultimately impairing cilia formation and endothelial cell integrity [12]. Chronic high‐fat diet‐induced accumulation of PA in the hypothalamus results in a significant reduction in both cilia number and cilia length in anorexigenic pro‐opiomelanocortin (POMC) neurons and other neuronal populations, thereby impairing insulin sensitivity [13]. This differential PA availability stems from cell‐type‐specific metabolism: endothelial cells actively convert PA via SCD1 and sequester it into lipid droplets, whereas hypothalamic neurons lack comparable clearance mechanisms, allowing PA to accumulate. Mechanistically, PA scarcity blocks palmitoylation in endothelia [12], whereas PA overload inhibits autophagy in neurons [13], and both conditions ultimately lead to cilia loss. Therefore, cells require precisely controlled levels of free PA to maintain cilia. Similarly, abnormal activation of fatty acid synthesis leads to the mislocalization of apical proteins in epithelial cells, such as glycoprotein 114/carcinoembryonic antigen (gp114/CEA), inhibiting primary cilium formation, thereby disrupting cell polarity and morphology [14]. In contrast, the microbiota‐derived short‐chain fatty acid butyrate promotes ciliogenesis by inhibiting histone deacetylases (HDACs), a factor for cilia disassembly and upregulating the expression of ciliogenic transcription factors, such as forkhead box j1 (Foxj1) and regulatory factor x 2/3 (Rfx2/3), leading to elongated primary cilia in hypothalamic agouti‐related peptide (AgRP) neurons, which contributes to anorexigenic effects and glucose homeostasis [15]. Collectively, these studies show that distinct fatty acids and lipid metabolites modulate cilia in terms of ciliary length, number, and signaling capability, enabling cilia to transmit metabolic cues to target tissues and thereby coordinate development or energy balance.

### 2.2 Lipid Distribution in Cilia

PtdIns is among the lipids that have been extensively investigated to exhibit a unique distribution pattern in cilia [16, 17]. Research indicates that phosphatidylinositol 4,5‐bisphosphate (PI(4,5)P_2_) and phosphatidylinositol 3,4,5‐trisphosphate (PI(3,4,5)P_3_) are primarily localized to the TZ on the inner leaflet of the membrane, forming a distinct ring‐shaped structure with a diameter of approximately 300 nanometers (nm) that encircles the axoneme. PI(4,5)P_2_ and PI(3,4,5)P_3_ occupy distinct subregions along the proximal‐distal axis within the TZ. PI(4,5)P_2_ is localized proximally, closer to the basal body, whilst PI(3,4,5)P_3_ occupies the distal region [18, 19, 20]. In contrast, phosphatidylinositol 4‑phosphate (PI4P) is localized to regions outside of the TZ toward the cilia tip direction (Figure 1) [21, 22, 23]. Another class, sphingolipids, with ceramide as their core structure, is also specifically enriched in the ciliary base membrane (Figure 2) [24]. During ciliogenesis in MDCK cells, which contain both the intracellular and extracellular ciliogenesis pathways, ceramides accumulate within the apical compartment of the basal body, nominated as the apical ceramide‐enriched compartment (ACEC), where they form ring‐shaped assemblies above or around the basal body [25]. Local enrichment of ceramides has also been observed in human embryonic kidney (HEK293T) cells, mouse astrocyte cilia, however, the ring‐shaped structure of ACEC was only reported in MDCK cells [25, 26]. PtdCho possesses a cylindrical molecular structure determined by the spatial arrangement of its choline head group and fatty acid tail chain. The two fatty acid chains of PtdCho are embedded within the hydrophobic core of the membrane via hydrophobic interactions, with chain length and saturation directly modulating membrane fluidity [5, 27]. Together with PtdCho, PtdEtn and PtdSer contribute to the regulation of membrane morphology. PtdEtn possesses a unique conical structure that contributes to the maintenance of ciliary membrane curvature. PtdSer possesses negatively charged glycerophospholipid head groups that participate in membrane protein distribution and signaling cascade regulation [28]. In cilia, PtdEtn and PtdSer are localized to the inner leaflet of the ciliary membrane [29, 30, 31]. The outer leaflet of the ciliary membrane contains inverted conical shaped lipids to maintain the membrane curvature at certain sites [30, 32]. Gangliosides (such as ganglioside M1), a subtype of glycosphingolipids, play a role in stabilizing membranes with high curvature at cilia base up to the tip, filling the outer phospholipid leaflet [33, 34]. The dynamic beating of motile cilia imposes substantial mechanical force on the ciliary membrane at the ciliary base, which critically requires gangliosides with long fatty acyl chains and large polar head groups to fill the outer phospholipid leaflet. A notable example is found in the flagellum of *Trypanosoma brucei*, in which gangliosides are highly abundant [35, 36]. Except for the contribution of PtdEtn and PtdSer to the uneven protein distribution among inner and outer leaflets, in mammalian cells, lipid asymmetry is maintained by P4‐ATPase flippases such as ATP11A and ATP11C, although their functional redundancy complicates the assignment of vital roles for each in cilia (Figure 1) [37, 38]. By contrast, the *Chlamydomonas* homolog aminophospholipid flippase 2 (ALA2) is clearly recruited to the ciliary membrane during ciliogenesis and is required for proper PtdEtn distribution and ciliary membrane curvature [30].

Given the highly dynamic nature of lipids in physiological activities and their instability during sample preparation, discrepancies in lipid localization have been widely reported. For example, ciliary PtdIns, particularly PI(4,5)P_2_, has yielded conflicting readouts regarding its relative abundance and distribution across the ciliary base, TZ, and shaft. Such inconsistencies arise predominantly from technical artifacts of detection [18, 39], although physiological lipid remodeling upon Hedgehog activation also contributes to divergence [20]. When fixed cells are permeabilized with methanol and imaged using a confocal microscope, PI(4,5)P_2_ shows signal not only in the TZ but also along the axoneme [20]. In contrast, when observed after permeabilization with Triton X‐100, PI(4,5)P_2_ is detected only in the TZ region; moreover, differences in microscopy techniques and reagents also resulted in significant variations in signal intensity within the TZ [21, 40]. When cells are treated with methanol and imaged using super‐resolution stimulated emission depletion (STED) microscopy, PI(4,5)P_2_ is specifically localized to the TZ [18]. Biases also exist between PtdIns antibodies and genetically encoded lipid biosensors [18, 39, 41, 42]. Fundamentally, the field has not yet reached a consensus on whether low‐level ciliary PI(4,5)P_2_ represents a physiological component or pathological lipid accumulation [18, 39]. Integrative strategies combining quantitative mass spectrometry, correlative light‐electron microscopy, and feasible AI for data integration and artifact correction, and absolutely calibrated lipid biosensors are therefore required to fully resolve this long‐standing controversy.

### 2.3 Lipid‐Protein Microdomains Distribution in Cilia

Lipid rafts, hereafter referred to as membrane microdomains or microdomains, are defined in this paper as regions enriched in cholesterol and sphingolipids that exhibit a liquid‐ordered structure [43]. Lipid rafts remain a subject of controversy. Although the liquid‐ordered phase can be clearly observed in artificial membrane systems, it cannot be directly visualized in living cells, and no consensus has been reached regarding its actual existence, size, and formation mechanisms in living cells [44, 45]. Nevertheless, ciliary membranes exhibit significantly greater enrichment of microdomains than plasma membranes, particularly at the ciliary base. Mechanistically, the saturated fatty acid chains of sphingolipids facilitate tight interactions with other lipid molecules, such as cholesterol, thereby promoting the formation of compact and stable microdomains [46]. Glycolipids reside in the outer leaflet of the microdomain phospholipid bilayer, with their sugar chains projecting toward the extracellular environment to participate in cell‐cell recognition [47]. Many proteins are anchored to the microdomains via glycosylphosphatidylinositol (GPI) or acylation modifications, such as palmitoylation and myristoylation (Figure 2) [48, 49]. Acylation not only enhances the affinity of proteins for the membrane but also promotes the confinement of signaling molecules within microdomains, thereby effectively amplifying and transmitting signals.

Among the lipid molecules present in microdomains, the cholesterol content of the ciliary membrane has long been a subject of debate. Early studies using filipin (a marker for non‐esterified cholesterol) reported that the ciliary membrane was enriched with free cholesterol [50, 51]. Using newly developed protein probes derived from lipid‐binding toxins (the non‐lytic Perfringolysin O (PFO) that specifically recognizes accessible cholesterol, Osterolysin A (OlyA) that recognizes sphingomyelin‐cholesterol complexes, and OlyA_E69A that recognizes total sphingomyelin), the researchers quantitatively compared the ciliary membrane with the adjacent plasma membrane and found that the sphingomyelin‐to‐cholesterol ratio in the ciliary membrane was significantly higher than that in the adjacent plasma membrane, with accessible cholesterol present at extremely low levels [52]. This finding showed that high levels of sphingomyelin sequester cholesterol into a state of low chemical activity. Inhibition of sphingomyelin synthesis releases the ‘chelated cholesterol’ as ‘accessible cholesterol’, thereby enhancing the regulation of the Hedgehog (Hh) signaling pathway [52].

Regarding the role of microdomains as gates or ferries for protein transport into cilia, previous studies have shown that the ciliary localization of certain proteins is indeed influenced by microdomains. For example, the ciliary localization of fibrocystin depends on microdomain distribution [53]. Cystin requires association with lipid rafts for its proper cilia localization; abrogation of this raft binding abolishes its ciliary targeting [54]. However, microdomains are not a prerequisite for the ciliary entry of all cilia proteins. For example, membrane‐localizing type III adenylyl cyclase (ACIII) and inositol polyphosphate‐5‐phosphatase E (INPP5E) can maintain normal ciliary localization even after lipid rafts are disrupted or cholesterol is stripped away [55]. This divergence arises from possibilities that different proteins utilize distinct entry routes into cilia (e.g., vesicular trafficking, diffusion, or IFT, which may confer varying degrees of microdomain dependence. Future studies should employ high‐resolution live‐cell imaging techniques and new lipid probes to systematically evaluate the specific impact of microdomains on the cilia proteins.

### 2.4 Roles of Lipids in Cilia Assembly/Disassembly Regulation

PtdSer, ceramide, PtdIns, and cholesterol all function in ciliogenesis. PtdSer behaves as one of the ‘molecular switches’ that initiate ciliary formation. PtdSer on the trans‐Golgi network and recycling endosome serves as a key recruitment signal by anchoring the regulatory protein Rabin8 to transport vesicles for targeting to the centrosome [56]. Ceramides are pivotal to ciliogenesis [25, 57]. Sphingomyelin phosphodiesterase 4 (SMPD4) generates ceramides through the degradation of sphingomyelin [58]. Mutations in SMPD4 cause cilia shortening or partial disassembly, whereas exogenous ceramide supplementation restores ciliary length [59]. *SMPD4* mutations are associated with neurological disorders such as cerebellar hypoplasia [59]. In cilia from mouse ependymal cells and *Chlamydomonas*, the ceramides interact with phosphor‐Tyr‐216‐glycogen synthase kinase‐3 (pYGSK3), resulting in the accumulation of pYGSK3 at the cilia base and tip. At the ciliary tip, pYGSK3 interacts with HDAC6 or triggers cargo release from motor protein complexes [9].

Centrosomal PI4P, which shows an inhibitory role in ciliogenesis, needs to be exhausted by type I gamma phosphatidylinositol phosphate kinase (PIPKIγ) to recruit positive ciliogenesis regulators such as tau tubulin kinase 2 (TTBK2) [60]. On the contrary, PI4P on the Golgi apparatus needs to be maintained by phosphatidylinositol 4‐kinase beta (PI4KB), which is imperative for the key factors such as Rab11a to be released from the Golgi and accumulate at centrioles for ciliogenesis [61]. Inside cilia, the polarized distribution of PI(4,5)P_2_ is critical for ciliary length regulation: an increase in distal PI(4,5)P_2_ induces vesicle shedding from the ciliary tip, thereby promoting subsequent cilia shortening or disassembly [62]; whereas PI(4,5)P_2_ deficiency blocks ciliary membrane endocytosis, while ongoing Golgi‐derived vesicle fusion facilitates the growth of the ciliary membrane or axoneme, resulting in a longer cilium [62].

Cholesterol not only fills the interstitial spaces between membrane lipids through its planar steroid ring structure, thereby maintaining membrane integrity and fluidity, but also exerts regulatory functions in ciliogenesis. The glycoprotein prominin‐1 (CD133) participates in regulating cell polarity, membrane topology, and interactions with the cytoskeleton [63]. Cholesterol promotes the ciliary targeting of CD133, enabling CD133 to regulate ARL13B and HDAC6 for cilia assembly [63]. Additionally, the role of cholesterol in regulating ciliogenesis or cilia length may be mediated through microdomains, which gather a dynamic pool of cholesterol.

In summary, lipids regulate ciliary assembly and disassembly by acting as both structural membrane components and signaling molecules that control protein recruitment, vesicle trafficking, and microdomain functions.

### 2.5 Role of Lipids in Cilia Motility

Lipid composition is related to the motility of cilia. Cholesterol and phospholipids play a role in regulating sperm flagella motility. Deficiency of Niemann‑Pick disease, type C2 (NPC2) reduces cholesterol content in the sperm flagellar membrane and alters membrane fluidity, disrupting motility [64]. Again in sperm flagella, PI(4,5)P_2_ is enriched along the ciliary membrane rather than being confined solely to the TZ, and this distribution may accommodate the rapid calcium signaling responses required for the regulation of ciliary motility [65]. Specifically, PI(4,5)P_2_ exhibits concentration in the midpiece region, which may also contribute to wave‐like flagellar motility in sperm [65]. In addition, the mitochondrial sheath assembly protein testis‐expressed protein 44 (TEX44) interacts with carnitine palmitoyltransferase 1B (CPT1B) to limit excessive oxidation of long‐chain fatty acids and prevent overproduction of reactive oxygen species. Loss of either TEX44 or CPT1B leads to incomplete mitochondrial sheath assembly and significantly reduced sperm motility, indicating that an imbalance in fatty acid oxidation can indirectly impair cilia motility [66]. Collectively, changes in membrane lipid composition affect motility in cilia and cause diseases [18, 65]. Likewise, in *Chlamydomonas* cells, ceramides in the flagella connect the membrane to the axoneme by binding to IFT particle, ceramide depletion widens the membrane‑axoneme gap, causes flagellar shortening and bulging, and severely impairs movement [11]. Collectively, these findings demonstrate that the impact of membrane lipids on cilia motility represents an evolutionarily conserved mechanism, and perturbations in lipid homeostasis can give rise to ciliary motility‐related disorders.

### 2.6 Roles of Lipids in Cilia‐Based Signaling Pathways

The Hh signaling pathway is a highly conserved cilia‐based signaling pathway [67, 68]. In mammals, Hh signaling involves a complex cascade of events within primary cilia, involving the G protein‐coupled receptor (GPCR) patched 1 (PTCH1), the sensor smoothened (Smo), and the downstream effector glioma‐associated oncogene (GLI) transcription factors [69]. PI4P promotes Smo activation and ensures its localization within cilia by directly binding to the Smo C‐terminus [70]. The binding drives two coupled key events: a conformational rearrangement of Smo andsubsequent phosphorylation of Smo. Both processes are essential for the stable accumulation of Smo within the primary cilium after the activation of the signaling pathway [70]. Cilia lacking INPP5E with a low amount of PI4P, abnormally accumulate the other GPCR GPR161, which negatively regulates the Hh signaling pathway by inhibiting signal transduction [42, 71]. Beyond Hh signaling‐related GPCRs, ciliary membrane PI4P also regulates the GPCR melanocortin 1 receptor (MC1R) in cilia [72]. Except for PI4P, cholesterol is a key regulatory lipid in the Hh pathway that can directly bind to and activate Smo (Figure 2) [73, 74, 75]. Smo contains multiple cholesterol‐binding domains, including the cysteine‐rich domain (CRD), the transmembrane domain (TMD), and the wedge‐shaped ligand domain (LD) situated between CRD and TMD [76, 77]. PTCH1, an inhibitor of Smo, exerts its function by binding cholesterol and reducing cholesterol levels on the membrane. Upon the binding of the Hh morphogen to PTCH1, cholesterol is released from PTCH1, thereby promoting Smo activation [77, 78]. Abnormal cholesterol levels disrupt Hh signaling in cilia. Peroxisomes transport cholesterol along microtubules to the ciliary membrane (Figure 2). Dysfunction of peroxisomes causes abnormal cholesterol levels in cilia and disrupts Hh signaling [79, 80]. Vertebrate olfactory receptors belong to the GPCR family, whereas receptors on the cilia of insect olfactory cells are ligand‐gated ion channels [81]. Research indicates that the distribution of PtdEtn and PtdSer within *Drosophila* olfactory cilia is crucial for their sensory function. dATP8B (a P4‐type ATPase) flippase is highly enriched in cilia and governs the asymmetric distribution of PtdEtn and PtdSer across membranes [31]. Deletion of *dATP8B* impairs *Drosophila* olfaction, indicating that the distribution of PtdEtn and PtdSer is essential not only for ciliary morphology but also for the physiological functions of the olfactory cells [31, 82]. During visual signal transmission, PtdEtn conjugated lipids directly supply the retinal aldehyde required for the rapid regeneration of rhodopsin for the outer segment (OS) of photoreceptors [83, 84]. Collectively, lipids govern the functions of membrane proteins such as GPCRs in cilia by fine‐tuning their critical biological functions for signaling.

## 3 Disruption of Lipid Homeostasis and Ciliopathies

Alterations in the lipid composition in cilia can compromise its fundamental role in harboring signaling proteins and maintaining proper signal transduction. Clinically, such alterations may contribute to a spectrum of genetic disorders, some of which are known as ciliopathies, which can affect the eyes, brain, kidneys, and other organs (Table 1). Here we summarize the updates in lipid‐related diseases.

**TABLE 1:** Pathological features of ciliopathies linked to ciliary lipid dysregulation.

Disease	Related genes	Abnormal lipids	Abnormal signaling pathway	Clinical phenotypes
ZS	*PEX1*, *PEX6*, *PEX10*, *PEX12*, *PEX26*, *PEX19*, *etc*. [137, 138, 139].	Cholesterol	Hh; disorders of peroxisomal biogenesis	Polycystic kidneys; pigmentary retinopathy; developmental delay; hepatic dysfunction, etc.
JBTS	*INPP5E*, *OFD1*, *TCTN2*, *CEP290*, *AHI1*, *TMEM67*, *RPGRIP1L*, *CPLANE1*, etc. [111].	PtdIns	Hh; olfactory signaling [140]	Cerebellar and brainstem malformations, producing a ‘molar tooth‐like structure’; intellectual disability, impaired balance; retinal dystrophy; cirrhosis of the liver; polydactyly, etc.
BBS	*BBS1*, *BBS2*, *ARL6*, *BBS4*, *BBS5*, *MKKS*, *BBS7*, *BBS8*, *BBS9*, *BBS10*, *BBS11*, *BBS12*, *BBS13*, *BBS14*, *BBS15*, *BBS16*, *BBS17*, *BBS18*, *BBS21*, *IFT57*, *IFT27*, *IFT74*, *IFT172*, *TSPOAP1*, *TTC21B*, etc. [89, 141, 142, 143, 144].	PtdIns; Cholesterol; Sphingolipids; Lipid rafts	Hh; Wnt/β‐catenin; fat differentiation‐related pathways	Retinal degeneration; obesity; polydactyly; renal abnormalities; intellectual disability, etc.
HLS	*HYLS1*, *KIF7*, *KIAA0586* [126, 145, 146].	PtdIns	Hh	Hydrocephalus in the fetus or newborn; polydactyly; cerebellar malformations; cleft palate, etc.
ADPKD	*PKD1*, *PKD2*, *IFT140*, etc. [147, 148].	Cholesterol	Polycystin ion channel signaling; EGFR pathway	Renal cyst; kidney failure.

### 3.1 Cholesterol‐Related Ciliopathies

#### 3.1.1 Bardet‐Biedl Syndrome (BBS)

BBS is an autosomal recessive disorder. The BBSome, a highly conserved multi‐subunit protein complex comprising eight conserved subunits, plays a pivotal role in maintaining ciliary function by acting as an adaptor for IFT complex [85]. Its core biological function involves balancing the localization of proteins and lipids between the plasma membrane and the ciliary membrane. Mutations in BBSome‐associated genes cause the classic manifestations of BBS, including retinal degeneration, obesity, polydactyly, renal abnormalities, and intellectual disability [86]. It is known that BBS has more than 20 disease‐causing genes, most of which encode the BBSome complex and IFT complex subunits [87], including IFT172, IFT74, and IFT27, among others (Table 1) [88, 89].

Emerging evidence highlights the critical relation of the BBSome and lipid homeostasis across different tissues and species [90, 91, 92]. In *Chlamydomonas bbs4* cilia, PtdEtn, which serves as a substrate for phospholipase D (PLD), decreases by approximately 30–50%, due to the ciliary accumulation of the lipase PLD [10]. In the OS of *zebrafish* photoreceptors, loss of *bbs1* results in significant accumulation of free cholesterol [93]. The impact of BBSomes on membrane lipid composition does not result from direct binding between BBSomes and membrane lipids, but rather depends on the cargo proteins controlled by BBSomes. Two mechanistic hypotheses can be proposed to explain the altered lipids in *bbs* cilia: on one hand, BBSomes or subunits affect the stability or the catalytic function of enzymes outside of cilia for lipid generation or delivery to cilia. On the other hand, BBSome deficiency may lead to the accumulation of enzymes such as lipases in cilia, thereby causing in situ changes of ciliary lipid composition.

Across species, non‐cilia proteins were observed to accumulate in cilia when BBSomes were absent. Studies in *Chlamydomonas* revealed that certain membrane‐associated proteins accumulate in BBSome‐deficient cilia, such as PLD and AMPK [94]. In *Lotmaria passim*, loss of BBS2 compromises microdomain or lipid raft integrity, resulting in the dissociation of two lipid raft‐associated proteins, LpFCaBP1N16 and LpFCaBP2N16, and consequently alters the lipid raft microenvironment [95]. In the OS of *zebrafish* photoreceptors, loss of *bbs1* results in significant accumulation of aberrant retention of membrane‐related proteins [93]. For mammals, accumulation of non‐OS proteins was also observed in the mouse *Bbs* model [93, 96]. For BBSome deficiency leading to the abnormal accumulation of membrane proteins or membrane‐associated proteins in cilia, we proposed the ‘Fish Trap’ model in which the TZ of the cilium acts as a unidirectional diffusion barrier, allowing membrane proteins to enter freely but preventing them from exiting; once these proteins enter the ciliary compartment, they become ‘trapped’ within the ciliary membrane and can only be retrieved through BBSome‐mediated active transport. BBSome cargos may possess features that enable their favorable interactions with BBSomes or via modifications like ubiquitination for their BBSome‐dependent ciliary exports [97]. Meanwhile, Fish Trap model may explain the cilia‐concentration of non‐BBSome cargos such as ARL13B and RAB34 for their steady ciliary localization [98, 99].

#### 3.1.2 Autosomal Dominant Polycystic Kidney Disease (ADPKD)

ADPKD and BBS both manifest with renal cyst formation, highlighting an intriguing convergence in underlying mechanisms (Table 1). The ciliary entry of polycystin‐1 (PC1) depends on the BBSome: the C‐terminus of PC1 interacts with BBSome subunits, while loss or pathogenic mutation of BBS1 or BBS3/ARL6 impairs the ciliary localization of PC1 [100]. Meanwhile, cholesterol directly binds polycystin‐2 (PC2), an interaction that is essential for the stable enrichment of PC2 in the primary ciliary membrane. Disruption of the cholesterol and PC2 interaction does not alter PC2 channel activity but prevents PC2 proper ciliary localization [101]. Moreover, the ciliary oxysterol 7β,27‐dihydroxycholesterol (7β,27‐DHC) acts as a key regulator of the polycystin ion channel complex, through binding to a cytoplasmic pocket formed by E208 and R581 on PC‑2 to gate the channel. Such dysregulations are closely associated with ADPKD [102]. Beyond cholesterol metabolism, dysregulated glycosphingolipid metabolism has emerged as an important contributor to PKD pathogenesis. Glycosphingolipids, particularly glucosylceramide (GlcCer) and lactosylceramide (LacCer), are markedly elevated in renal tissues and cells from PKD patients [103, 104, 105]. GlcCer and LacCer accumulation may promote cyst initiation and growth by altering the activity of ion channels within lipid rafts in cilia [106].

#### 3.1.3 Zellweger Syndrome (ZS)

ZS is an autosomal recessive disorder defined by disturbed cholesterol homeostasis, frequently manifesting with polycystic kidneys, pigmentary retinopathy, developmental delay, and hepatic dysfunction (Table 1) [80, 107]. While mammalian cells normally acquire cholesterol through de novo synthesis in the endoplasmic reticulum and receptor‐mediated endocytosis of low‐density lipoprotein, peroxisomes play a critical role in intracellular transport. Normally, peroxisomes undergo directional transport along microtubules to deliver cholesterol to ciliary pocket membrane contacts for local release [80]. In the absence of functional peroxisomes, cholesterol abnormally accumulates within the cytoplasm [108]. In ZS, the systemic loss of peroxisomes disrupts intracellular cholesterol trafficking, impairing proper formation and function of cilia across diverse cell types. The resulting cholesterol deficit at the ciliary membrane disrupts essential signaling pathways, such as the Hh pathway, thereby driving the pathogenesis of ZS [109]. Currently, there is still no cure for ZS in clinical practice; only symptomatic and supportive treatment is available [107]. Although new therapies such as gene editing have shown preliminary progress in animal studies, they have not yet entered clinical use [110].

### 3.2 Phosphatidylinositol Disruption‐Related Ciliopathies

#### 3.2.1 Joubert Syndrome (JBTS)

JBTS is an autosomal recessive genetic disorder. Clinically, it is typically characterized by specific malformations of the cerebellum and brainstem, producing the characteristic ‘molar tooth‐like’ structure (Table 1) [111]. Affected individuals exhibit intellectual disability, impaired balance, retinal dystrophy, liver cirrhosis, and polydactyly [112]. Notably, congenital ocular motor apraxia (COMA) is an early clue for JBTS (∼52% of patients show ‘molar tooth’ sign), and atypical hypertonia can occur, emphasizing phenotypic diversity and the need for multidisciplinary assessment [113]. Mutations in the *INPP5E* are associated with JBTS [114, 115]. Dysfunction of INPP5E leads to markedly elevated levels of PI(4,5)P_2_ and PI(3,4,5)P_3_ within cilia [115]. PI(4,5)P_2_ plays a crucial role in recruiting the ciliary cargo carrier protein TULP3, a member of the TUBBY family that associates with the IFT system. TULP3 binds to PI(4,5)P_2_ via its C‐terminal TUBBY domain, and this interaction enhances the affinity of TULP3 for the ciliary membrane and facilitates the transport of membrane protein cargo into the cilium [116, 117, 118, 119]. Disrupted inositol phosphate homeostasis impairs the localization and activity of key proteins in the Hh signaling pathway, such as Smo and GLI. Ultimately, dysfunction of this signaling pathway results in characteristic neurodevelopmental abnormalities and multi‐organ defects [120, 121]. The ciliary targeting of INPP5E is coordinately regulated by four conserved ciliary localization signals (CLS1‐CLS4) on INPP5E. Although these CLS exhibit partial functional redundancy, each plays distinct roles in recruiting specific molecular partners: CLS4 recruits PDE6D (phosphodiesterase 6D), RPGR (retinitis pigmentosa GTPase regulator), and ARL13B; CLS2‐CLS3 modulate binding to TULP3 (TUB‐like protein 3), ARL13B, and CEP164 (centrosomal protein 164); and CLS1 together with CLS4 cooperatively mediates the interaction with ATG16L1 (autophagy related 16 like 1) [122]. Notably, mutations in the same gene, *INPP5E*, can lead to diverse phenotypes and different diseases. For instance, JBTS predominantly affects the cerebellum and retina, whereas MORM syndrome is characterized by obesity and intellectual disability [123]. This phenotypic discrepancy may arise from distinct molecular defects caused by different *INPP5E* mutations: JBTS‐linked catalytic‐domain missense variants primarily impair phosphatase activity while preserving ciliary targeting [115, 122], whereas MORM‐associated C‐terminal truncation impairs CLS4‐dependent ciliary anchoring while retaining enzymatic activity [122, 124]; thus, disease outcomes may depend on whether a given mutation predominantly compromises INPP5E catalytic function or its ciliary‐targeting machinery and associated proteins.

#### 3.2.2 Hydrolethalus Syndrome (HLS)

HLS is a severe autosomal recessive ciliopathy that presents with prenatal or neonatal hydrocephalus, polydactyly, cerebellar malformations, and cleft palate, reflecting widespread neurodevelopmental defects (Table 1) [125]. The disease is primarily caused by loss‐of‐function mutations in the conserved centrosomal protein HYLS1, including a disease‐associated substitution of a highly conserved aspartic acid residue with glycine that impairs HYLS1 function [126, 127]. Mechanistically, HYLS1 is recruited to the ciliary base through interaction with PIPKIγ, where HYLS1 relieves the autoinhibitory dimerization of PIPKIγ and thereby promotes PIPKIγ activation to remove PI4P for ciliogenesis [60]. In addition, HYLS1 contributes to ciliary signaling by modulating Hh pathway activity; in HYLS1 (D211G) mutant cells, the mutant protein fails to anchor nephronophthisis (NPHP) at the TZ, which is required for β‐arrestin translocation, without this step, GPR161 cannot be removed from cilia upon Smo agonist (SAG) stimulation, resulting in suppressed Hh signaling [60]. Thus, HYLS1 integrates centrosomal organization, ciliary lipid remodeling, and signal transduction, providing a mechanistic basis for the severe developmental phenotypes associated with HLS.

Above all, the crosstalk among lipids in ciliopathies further underscores the broad significance of ciliary membrane lipid homeostasis in health conditions, highlighting the significance of the lipid microenvironment as a therapeutic target for multiple ciliary disorders.

## 4 Advances in Lipid‐Targeted Therapies for Ciliopathy

Significant progress has been made in the development of drugs targeting lipid metabolism for ciliopathies. Glucosylceramide synthase (GCS) is a key enzyme in the de novo synthesis of glycosphingolipids, and its inhibitors have demonstrated clear therapeutic efficacy. For example, in the mouse model of BBS, the GCS inhibitor Genz‐667161 significantly alleviated obesity, retinal degeneration, and olfactory deficits, with these beneficial effects being associated with the restoration of ciliary structure and Hh signaling pathway activity [128]. Beyond GCS inhibition, other therapeutic approaches have also been explored for BBS. The approved melanocortin‐4 receptor (MC4R) agonist setmelanotide significantly reduces hyperphagia and body weight and improves metabolic dysfunction‐associated steatotic liver disease (MASLD) and renal function [129]. However, its occurrence of skin hyperpigmentation and rare reports of dysplastic nevi or even melanoma, necessitates routine dermatologic monitoring [130]. Semaglutide, a GLP‐1 receptor agonist, has likewise shown weight‐loss and metabolic benefits in individual cases of BBS; however, its current evidence base is limited to case reports and lacks support from large‐scale studies [131]. Therefore, setmelanotide remains the core targeted therapy for BBS. In addition to pharmacological interventions, molecular therapies targeting the genetic basis of BBS are also being explored. For recessive exon‐splicing defects caused by *BBS16* mutations, antisense oligonucleotide (ASO) therapy has achieved partial recovery of protein expression in preclinical models. Specifically, following entry into the nucleus, ASOs bind to target sequences within precursor mRNA, thereby preventing aberrant splicing. This approach provides a new strategy for personalized gene correction therapy for BBS [132]. In the context of ADPKD, the therapeutic potential of targeting glycosphingolipid metabolism has also been investigated. In preclinical studies, the GCS inhibitor Venglustat was shown to slow cyst progression [103]. Notably, the ketogenic diet represents another metabolic intervention capable of influencing lipid metabolism through the induction of ketone body production. Clinical trials of the ketogenic diet (KETO‐ADPKD) demonstrated that patients were able to adhere to the ketogenic diet, which significantly improved estimated glomerular filtration rate and reduced liver volume [133]. As *INPP5E* is one of the causative genes for Joubert syndrome, the PI3K inhibitor LY294002 reduces PI(3,4,5)P_3_ levels and partially rescues ciliary defects and renal cyst phenotypes in *inpp5e*‑knockdown *zebrafish* [134]. PI3K inhibitors may serve as an indirect regulatory strategy targeting downstream of INPP5E. Nevertheless, small‑molecule agents that directly target INPP5E have not yet been reported [135]. The few compounds reported to exhibit INPP5E inhibitory activity are broad‐spectrum pan‐inhibitors with significant cytotoxicity, lacking selectivity among the 5‐phosphatase family [136]. Future therapeutic strategies targeting lipids in ciliopathies may be pursued from the novel detecting technologies, basic mechanistic research, and translational therapy.

## 5 Perspectives

Therefore, future research on ciliary membrane lipid research can focus on three frontiers. First, cryo‐EM and in situ structural biology can now resolve lipid‐protein complexes at atomic resolution, revealing how disease mutations disrupt ciliary protein regulation. Second, the bidirectional crosstalk between ciliary lipids and systemic metabolism warrants deeper investigation: cilia sense or reflect the metabolic cues and cilia dysfunction may be related to obesity and dyslipidemia, offering opportunities for lipid‐based therapeutics. Third, lipid rafts/microdomains in cilia, which can be drug carriers, demand dynamic mapping; spatial lipidomics and super‐resolution imaging can define their assembly and signaling modules, guiding targeted ciliopathy therapies.

## Author Contributions

**Zhenzhou Huang**: writing – original draft, writing – review and editing, conceptualization. **Yuan‐yuan Liu**: writing – original draft. **Hanqi Sun**: investigation. **Peiwei Liu**: writing – original draft, writing – review and editing, conceptualization, supervision, funding acquisition.

## Funding

This work was supported by grants from the National Natural Science Foundation of China (32270808 to P. Liu), Taishan Scholar Foundation of Shandong Province (tsqnz20231208 to P. Liu), and the Department of Science and Technology of Shandong Province (2024HWYQ‐052 to P. Liu).

## Conflicts of Interest

The authors declare no conflicts of interest.

## Data Availability

The data that supports the findings of this study are available in the supplementary material of this article.
